# Association Between Long-Term HbA1c Variability and Functional Limitation in Individuals Aged Over 50 Years: A Retrospective Cohort Study

**DOI:** 10.3389/fendo.2022.847348

**Published:** 2022-04-29

**Authors:** Di Shao, Shuang-Shuang Wang, Ji-Wei Sun, Hai-Peng Wang, Qiang Sun

**Affiliations:** ^1^ Centre for Health Management and Policy Research, School of Public Health, NHC Key Laboratory of Health Economics and Policy Research, Cheeloo College of Medicine, Shandong University, Jinan, China; ^2^ Center for Reproductive Medicine, School of Medicine, Cheeloo College of Medicine, Shandong University, Jinan, China; ^3^ Key Laboratory of Reproductive Endocrinology of Ministry of Education, Shandong University, Jinan, China

**Keywords:** functional limitation, glycated hemoglobin A1c, glycemic variability, HbA1c, mobility, physical functioning

## Abstract

**Background:**

As mean HbA1c provides incomplete information regarding glycemic variability, there has been considerable interest in the emerging association between glycemic variability and macrovascular events and with microvascular complications and mortality in adults with and without diabetes. However, the association between long-term glycemic variability, represented by visit-to-visit HbA1c variability, and functional limitations has not been clarified in previous literature. The present study aimed to explore the longitudinal association between long-term glycemic variability, represented by visit-to-visit HbA1c variability and functional limitations.

**Methods:**

This cohort study included adults aged over 50 years who participated in the 2006 to 2016 waves of the Health and Retirement Study. Physical functions, including mobility, large muscle function, activities of daily living (ADLs), and instrumental ADLs (IADLs), were assessed at baseline and every 2 years, and HbA1c levels were assessed at baseline and every 4 years. Visit-to-visit HbA1c variability was calculated using the HbA1c variability score (HVS) during the follow-up period. Generalized estimating equation models were used to evaluate the longitudinal association between HbA1c variability and functional limitations with adjustment for a series of confounders.

**Results:**

A total of 5,544 participants having three HbA1c measurements from 2006 to 2016, having two or more physical function measures (including one at baseline), and age over 50 years were included in this analysis. The mean age at baseline was 66.13 ± 8.39 years. A total of 916 (16.5%) participants had an HVS = 100, and 35.1% had an HVS = 50. The highest HVS category (HVS =100) was associated with increased functional status score (β = 0.093, 95% CI: 0.021–0.165) in comparison with the lowest HVS category (HVS = 0). Sensitivity analyses using the CV and SD of HbA1c as measures of variability showed similar associations between HbA1c variability and functional limitation. An incremental increase in HbA1c-CV (β = 0.630, 95% CI: 0.127–1.132) or HbA1c-SD (β = 0.078, 95% CI: 0.006–0.150) was associated with an increase in functional limitation in the fully adjusted model.

**Conclusions:**

HbA1c variability was associated with heightened difficulty in performing functional activities over time after adjusting for mean HbA1c levels and multiple demographics and comorbidities. This study provides further evidence regarding the detrimental effect of HbA1c variability and highlights the significance of steady glycemic control.

## Introduction

Physical functioning is a multidimensional concept encompassing mobility, large muscle functioning, gross motor skills, fine motor skills, and the ability to perform activities of daily living (ADLs) and instrumental ADLs (IADLs) (1–3). It is an essential aspect of daily life and enables autonomy and participation in meaningful physical, social, and cultural activities. Limitation of physical functioning threatens independence and is an independent risk factor for impaired quality of life, institutionalization, further functional decline, and premature mortality in older adults (3–5). Accordingly, identification of risk factors for physical function limitations in middle-aged and elderly individuals may provide insights into appropriate clinical practice and public health interventions to inform optimal self-management and clinical management of adults with these conditions (2, 3).

Hemoglobin A1c (HbA1c) is the current gold standard for monitoring blood glucose control and is now recommended for use in diagnosing diabetes and identifying individuals at risk of developing diabetes (6). The association between diabetes and functional limitation and disability is well documented in literature (7–10). Previous population-based longitudinal studies have also indicated that impaired fasting glucose, impaired glucose tolerance, and newly diagnosed diabetes are associated with reduced of health-related functioning, and that this is evident before the onset of these conditions (11–13). People with insulin resistance, and as they age, they are significantly more likely to have a deterioration in their quality of life in the areas of physical functioning, emotional role limitations, social functioning, pain and general health perception (14). Evidence for the association between diabetes management using glycemic markers and physical function limitations has been inconsistent. Some studies have reported that poor glycemic control is associated with decreased physical function, and others have indicated a significant association between tight (lower) glycemic control and physical disability; however, some studies reported that there was no significant association (15–17). A prospective cohort study indicated a nonmonotonic longitudinal relationship between HbA1c levels and the physical functioning decline in later life, however the HbA1c was assessed only at baseline (18).

In this context, whether an average glycemic measure is most appropriate for assessing the risk of complications is currently under debate. The concept of glycemic variability, which is related to fluctuations in glycemia, has recently emerged as another measure of glycemic control, which might constitute an additive or even better predictor of diabetic complications compared to mean HbA1c levels (19). Two components of glycemic variability have been recognized: short-term glycemic variability over days to weeks, and long-term glycemic variability ascertained by calculating visit-to-visit fluctuations of HbA1c over periods of follow-up lasting months to years (19). Although it remains controversial, some reviews and meta-analyses have shown significant associations between HbA1c variability and all-cause mortality, renal disease, and cardiovascular disease in type 2 diabetes and retinopathy, renal disease, and cardiovascular disease in type 1 diabetes (20–22).

There is also considerable interest in the emerging association between glycemic variability and decline in cognitive function and the increased level of symptoms of depression (23, 24). However, most of the included studies had limitations such as little adjustment for key confounders, concentration on secondary care patients with diabetes, and high levels of heterogeneity between studies, possibly related to different definitions and measurements of variability (20). Furthermore, glycemic variability seems to have an effect in individuals without diabetes (19, 25). A study including 6,756 individuals without diabetes indicated an association between high HbA1c variability and increased risks of incident major adverse cardiovascular events and death from all causes (25).

In conclusion, although some studies have examined HbA1c and its association with functional disability, these studies examined cross-sectional data or assessed functional decline over a brief period of time and have reported controversial results (16–18, 26). As mean HbA1c provides incomplete information regarding glycemic variability, there has been considerable interest in the emerging association between visit-to-visit glycemic variability and macrovascular events and with microvascular complications and mortality in adults with and without diabetes (19). However, the breadth of information on the longitudinal association between HbA1c variability and functional limitations is limited. Whether glycemic variability in individuals without diabetes is an independent risk factor for functional limitation is currently unknown.

This study aimed to determine the association between HbA1c variability and functional limitations across a wide range of physical tasks, after accounting for a series of sociodemographic confounders and comorbidities, in a nationally representative sample of middle-aged and elderly adults. We hypothesized that a higher variability in HbA1c, represented as the higher HbA1c variability score (HVS) and the intra-individual SD and coefficient of variation (CV) of HbA1c value across visits, would be associated with more difficulties in performing functional activities in this population after adjustment for potential confounders and mean HbA1c.

## Materials And Methods

### Data Source and Study Population

We used data from the 2006 to 2016 waves of the Health and Retirement Study (HRS) (27). The HRS is a longitudinal cohort study of health and retirement among American adults aged 50 years and older which collects data on demographics, socioeconomic factors, health conditions, and behavioral indicators biennially. HRS began to collect dried blood spot (DBS) blood-based biomarkers from half of the sample population in 2006, and the other half of the population provided DBS biomarker data in 2008 (28). The first group provided blood samples again in 2010 and 2014, and the second group provided repeat blood samples in 2012 and 2016, creating a 4-year interval between the biomarker blood collections. The time of the first HbA1c measurement was considered the baseline for all participants.

The RAND HRS Longitudinal File is an easy-to-use dataset based on the HRS core data, and it was used for analyses (29, 30). This file was developed at RAND with funding from the National Institute on Aging and the Social Security Administration. The HRS is sponsored by the National Institute on Aging (grant number NIA U01AG009740) and is conducted by the University of Michigan.

### Inclusion and Exclusion Criteria

The inclusion criteria for this study included the following: participants having three HbA1c measurements from 2006 to 2016, having two or more physical function measures (including one at baseline), and age over 50 years. A total of 5,796 respondents who had three HbA1c measurements from 2006 to 2016 were identified, after excluding those with missing physical function measures (n=48) and those aged less than 50 years (n=204), a total of 5,544 participants were included in this analysis.

### Measurement of HbA1c and HbA1c Variability

Blood sample collection and HbA1c measurement in the HRS were conducted every 4 years. The details of this process have been described elsewhere (28). The National Health and Nutrition Examination Survey-equivalent assay values of HbA1c in the HRS were used for analysis in our study, as recommended (28). Mean HbA1c values were calculated based on mean values of all visits for each participant. To better fit clinical practice, the visit-to-visit variability in HbA1c was defined as the HbA1c variability score (HVS), calculated by the number of successive measurements which differed by 0.5% (5.5 mmol/mol) or more divided by the number of comparisons and then multiplied by 100 (31, 32). Due to the lack of an appropriate gold-standard measurement for HbA1c variability, we calculated two other metrics, including the intra-individual SD and the coefficient of variation (CV) across visits as additional measures of glycemic variability (19).

### Functional Limitation

Physical function was assessed every 2 years in the HRS (33). We used several summary measures, including measures for mobility, large muscle function, ADLs, and IADLs for functional limitations located in the biennial core interview (34). Physical function was measured using 17 distinct physical tasks derived from well-validated questionnaires and were categorized into four functional domains according to published definitions: mobility (five tasks, namely, walking one block, walking several blocks, walking across a room, climbing one flight of stairs, and climbing several flights of stairs), large muscle limitation (four tasks, namely, sitting for 2 h, getting up from a chair, stooping or kneeling or crouching, and pushing or pulling a large object), ADLs (ADLs, three tasks, namely, bathing, eating, and dressing), and IADLs (IADLs, five tasks, namely, using a telephone, taking medication, handling money, shopping, and preparing meals) (33, 34).

For each task, a code of 0 indicated that the respondent did not report any problems with the activity. A code of 1 indicated that the respondent reported some difficulty with the activity or could not perform the activity. We used a composite score of the 17 items summed to obtain a disability score, with higher scores indicating greater disability (range, 0–17). This composite measure, which captures a broad range of disability from early or “preclinical” disability to later personal care disability, has the advantage of capturing finer graduations in limitations and reducing ceiling or flooring effects (33, 35).

### Demographic and Clinical Covariates

Covariates shown by previous studies to be associated with HbA1c levels and physical function were selected for analyses. The demographic covariates included age (continuous variable), sex (male or female), race (Hispanic, non-Hispanic White, non-Hispanic Black, others), marital status (married or partnered, separated or divorced, widowed, never married), and current smoking (yes or no). Body mass index (BMI) was calculated as weight in kilograms divided by height in meters squared (kg/m^2^) and treated as a continuous variable. Depressive symptoms were measured using an 8-item version of the Center for Epidemiologic Studies Depression Scale, with higher scores indicating more depressive symptomology (36). Cognitive function included immediate and delayed word recall, the serial 7s test, counting backward, naming tasks (e.g., date naming), and vocabulary questions, resulting in a score range of 0–35 (37). Comorbidities included dichotomous measures (yes or no) of self-reported physician’s diagnosis of (1) high blood pressure or hypertension; (2) diabetes or high blood sugar; (3) cancer or a malignant tumor of any type except skin cancer; (4) chronic lung disease other than asthma, such as chronic bronchitis or emphysema; (5) heart problems; (6) stroke or transient ischemic attack; (7) emotional and nervous disorders or psychiatric problems; and (8) arthritis or rheumatism (30). Sex and race were adjusted using baseline data, whereas other confounder variables were included as time-variants and adjusted using multi-wave data.

### Statistical Analyses

Continuous variables are described using median values (lower and upper quartiles), and categorical variables are presented as numbers (proportions). HVS was calculated as a measure of glycemic variability. Participants were grouped in terms of HVS, and baseline characteristics were compared using Kruskal-Wallis H test or Pearson χ^2^ tests, when appropriate.

Generalized estimating equations with a negative binomial distribution and an unstructured covariance matrix were used to evaluate the longitudinal association between the long-term HbA1c variability and functional status. Negative binomial regression was used to account for the over-dispersion of the functional scores. The effect of variability in HbA1c was calculated by modeling the HVS as a category variable (0, 50, 100, with HVS=0 as reference). Model 1 was adjusted for age, sex, race, and marital status, and mean HbA1c value. Model 2 was additionally adjusted for current smoking status, BMI, depression, cognitive function, hypertension, diabetes, cancer, lung disease, heart disease, stroke, arthritis, and psychiatric disorders. Sensitivity analyses were performed for outcome by using the SD and CV of the HbA1c instead of the HVS. To examine potential modification effects, interactions between HVS and age, sex and BMI were investigated. Whenever there was evidence of interaction (p < 0.05 for interaction term), stratified analyses were performed.

All significance tests were two-tailed, and a p-value of less than 0.05 was considered to indicate statistical significance. Statistical analysis was performed using SAS 9.4 software.

## Results

### Baseline Demographics and Clinical Characteristics

Mean age at baseline was 66.13 ± 8.39 years. A total of 3,349 (60.41%) participants were women. The participants were categorized into three groups according to HbA1c variability score (HVS = 0, 50, 100). Thirty-five percent of the patients had an HVS = 50; 16.5% had an HVS = 100. **Table 1** presents the characteristics of the included participants across the HbA1c variability score categories. There were significant differences in baseline characteristics, including race, marital status, BMI, depression symptoms, cognitive function, self-reported doctor diagnosis of diabetes, hypertension, heart disease, arthritis, and psychiatric disorder between groups (*p* s < 0.05).

**Table 1:** Baseline Characteristics of adults aged over 50 years of age by HbA1c variability score (N=5544).

Characteristics	Total	HbA1c variability score (HVS) categories			*H* or *χ*2	*p*
	N = 5544	0 (n = 2683)	50 (n = 1945)	100 (n = 916)		
Range of HbA1c CV	0.05 (0.03, 0.08)	0.03 (0.02, 0.04)	0.07 (0.06, 0.10)	0.11 (0.08, 0.15)	3900.063	**<0.001**
Range of HbA1c SD	0.31 (0.19, 0.49)	0.18 (0.13, 0.24)	0.43 (0.34, 0.56)	0.70 (0.50, 1.05)	3989.302	**<0.001**
HbA1c mean,%	5.69 (5.41, 6.11)	5.56 (5.35, 5.81)	5.77 (5.46, 6.26)	6.29 (5.71, 7.19)	746.676	**<0.001**
Age (years)	66.00 (59.00,72.00)	66.00 (59.00,72.00)	66.00 (59.00,72.00)	66.00 (59.00,72.00)	1.332	0.514
Sex, n (%)					1.406	0.495
Male	2195 (39.59)	1041 (38.80)	787 (40.46)	367 (40.07)		
Female	3349 (60.41)	1642 (61.20)	1158 (59.54)	549 (59.93)		
Race, n (%)					107.038	**<0.001**
Hispanic	440 (7.94)	193 (7.19)	143 (7.35)	104 (11.35)		
Not Hispanic white	4325 (78.01)	2201 (82.04)	1508 (77.53)	616 (67.25)		
Not Hispanic black	651 (11.74)	222 (8.27)	260 (13.37)	169 (18.45)		
Not Hispanic other	128 (2.31)	67 (2.50)	34 (1.75)	27 (2.95)		
Marital status, n (%)					26.588	**<0.001**
Married or partnered	3768 (67.97)	1894 (70.59)	1294 (66.53)	580 (63.32)		
Separated or divorced	753 (13.58)	353 (13.16)	261 (13.42)	139 (15.17)		
Widowed	841 (15.17)	370 (13.79)	315 (16.20)	156 (17.03)		
Never married	182 (3.28)	66 (2.46)	75 (3.86)	41 (4.48)		
Current smoker, n (%)	630 (11.45)	291 (10.94)	231 (11.95)	108 (11.88)	1.327	0.515
BMI, kg/m^2^	29.00 (25.60,32.90)	28.30 (25.10,32.00)	29.10 (25.90,33.20)	30.50 (27.10,34.80)	106.941	**<0.001**
Depression symptoms	0.00 (0.00, 2.00)	0.00 (0.00, 2.00)	0.00 (0.00, 2.00)	1.00 (0.00, 2.00)	32.335	**<0.001**
Cognition	24.00 (21.00, 26.00)	24.00 (21.00, 26.00)	24.00 (21.00, 26.00)	23.00 (20.00, 26.00)	14.465	**0.001**
Diabetes, n (%)	951 (17.17)	192 (7.16)	385 (19.82)	374 (40.83)	559.021	**<0.001**
Hypertension, n (%)	2910 (52.53)	1278 (47.65)	1061 (54.63)	571 (62.34)	64.376	**<0.001**
Cancer, n (%)	653 (11.80)	316 (11.80)	244 (12.57)	93 (10.18)	3.427	0.180
Lung disease, n (%)	366 (6.61)	171 (6.38)	125 (6.44)	70 (7.66)	1.956	0.376
Heart disease, n (%)	1013 (18.29)	449 (16.75)	373 (19.18)	191 (20.90)	9.415	**0.009**
Stroke, n (%)	275 (4.96)	123 (4.59)	103 (5.30)	49 (5.35)	1.561	0.458
Arthritis, n (%)	3158 (56.97)	1484 (55.31)	1117 (57.46)	557 (60.81)	8.705	**0.013**
Psychiatric disorder, n (%)	742 (13.40)	321 (11.99)	278 (14.31)	143 (15.63)	9.904	**0.007**
Functional total score	1.00 (0.00, 3.00)	1.00 (0.00, 3.00)	1.00 (0.00, 3.00)	2.00 (0.00, 4.00)	51.272	**<0.001**

Values are median (lower quartile, upper quartile) for continuous variables and number (%) for continuous variables. *Calculated using Kruskal-Wallis H test or Pearson χ2 test. p values less than .05 are in bold.

### Association Between HbA1c Variability and Functional Limitations

**Table 2** provides the results of the generalized estimating equation models to understand the influence of glycemic variability on functional limitations. After adjusting for demographics, in comparison with the reference (lowest HVS category, HVS =0), the highest HVS category (HVS =100) and medium HVS category (HVS =50) were associated with increased functional limitation score (β = 0.168, 95% CI: 0.089–0.247; β = 0.102, 95% CI: 0.041–0.162) in model 1. In the fully adjusted model, the highest HVS category was associated with increased functional status score (β = 0.093, 95% CI: 0.021–0.165) in comparison with the lowest HVS category.

**Table 2:** Association between HbA1c variability and functional limitation using GEE models (N=5544).

		β	SE	z	*p*	95% CI lower	95% CI Upper	OR	95% CI lower	95% CI Upper
**Model 1**	Intercept	-2.011	0.156	-12.92	**<0.001**	-2.315	-1.705	0.134	0.099	0.182
	HVS=100	0.168	0.040	4.18	**<0.001**	0.089	0.247	1.183	1.093	1.280
	HVS=50	0.102	0.031	3.29	**0.001**	0.041	0.162	1.107	1.042	1.176
	HVS=0	Ref.								
	HbA1c Mean	0.153	0.017	8.99	**<0.001**	0.120	0.186	1.165	1.127	1.204
	Age	0.028	0.001	20.26	**<0.001**	0.025	0.030	1.028	1.025	1.030
	Female	0.332	0.029	11.34	**<0.001**	0.275	0.390	1.394	1.317	1.477
	Male	Ref.								
	Not Hispanic Other	-0.041	0.117	-0.35	0.723	-0.270	0.187	0.960	0.763	1.206
	Not Hispanic Black	-0.013	0.058	-0.21	0.830	-0.126	0.101	0.987	0.882	1.106
	Not Hispanic White	-0.266	0.048	-5.59	**<0.001**	-0.359	-0.173	0.766	0.698	0.841
	Hispanic	Ref.								
	Never Married	0.111	0.053	2.08	**0.038**	0.006	0.215	1.117	1.006	1.240
	Widowed	0.042	0.022	1.88	0.061	-0.002	0.086	1.043	0.998	1.090
	Separated or divorced	0.169	0.031	5.49	**<0.001**	0.109	0.229	1.184	1.115	1.257
	Married or partnered	Ref.								
**Model 2**	Intercept	-2.218	0.219	-10.12	**<0.001**	-2.648	-1.789	0.109	0.071	0.167
	HVS=100	0.093	0.037	2.53	**0.011**	0.021	0.165	1.097	1.021	1.179
	HVS=50	0.050	0.028	1.82	0.069	-0.004	0.104	1.051	0.996	1.110
	HVS=0	Ref.								
	HbA1c Mean	-0.016	0.020	-0.82	0.414	-0.056	0.023	0.984	0.946	1.023
	Age	0.024	0.002	12.96	**<0.001**	0.020	0.027	1.024	1.020	1.027
	Female	0.225	0.028	8.14	**<0.001**	0.171	0.279	1.252	1.186	1.322
	Male	Ref.								
	Not Hispanic Other	-0.087	0.109	-0.79	0.427	-0.301	0.127	0.917	0.740	1.135
	Not Hispanic Black	-0.141	0.055	-2.56	**0.011**	-0.249	-0.033	0.868	0.780	0.968
	Not Hispanic White	-0.164	0.046	-3.56	**0.004**	-0.255	-0.074	0.849	0.775	0.929
	Hispanic	Ref.								
	Never Married	0.069	0.069	1.00	0.317	-0.066	0.205	1.071	0.936	1.228
	Widowed	-0.053	0.028	-1.89	0.059	-0.107	0.002	0.948	0.899	1.002
	Separated or divorced	0.050	0.037	1.33	0.183	-0.024	0.123	1.051	0.976	1.131
	Married or partnered	Ref.								
	Current smoker	0.207	0.042	4.92	**<0.001**	0.124	0.289	1.230	1.132	1.335
	BMI	0.035	0.002	18.20	**<0.001**	0.031	0.039	1.036	1.031	1.040
	Depression	0.132	0.005	24.29	**<0.001**	0.122	0.143	1.141	1.130	1.154
	Cognition	-0.022	0.003	-8.14	**<0.001**	-0.027	-0.017	0.978	0.973	0.983
	Diabetes	0.114	0.032	3.62	**<0.001**	0.053	0.176	1.121	1.054	1.192
	Hypertension	0.058	0.027	2.14	**0.032**	0.005	0.111	1.060	1.005	1.117
	Cancer	0.073	0.028	2.60	**0.009**	0.018	0.128	1.076	1.018	1.137
	Lung disease	0.298	0.032	9.45	**<0.001**	0.236	0.360	1.347	1.266	1.433
	Heart disease	0.205	0.024	8.45	**<0.001**	0.158	0.254	1.228	1.171	1.289
	Stroke	0.225	0.037	6.10	**<0.001**	0.152	0.297	1.252	1.164	1.346
	Arthritis	0.614	0.034	18.20	**<0.001**	0.548	0.681	1.848	1.730	1.976
	Psychiatric disorder	0.143	0.030	4.70	**<0.001**	0.083	0.202	1.154	1.087	1.224

HVS, HbA1c variability score. Model 1, adjusted for age, sex, race, marital status, and mean HbA1c; Model 2, further adjusted for current smoking, BMI, depressive symptoms, cognition, hypertension, diabetes, cancer, lung disease, heart disease, stroke, arthritis and psychiatric disorder. p values less than .05 are in bold.

An incremental increase in the mean HbA1c value was associated with an increased functional status score (β = 0.153, 95% CI: 0.120–0.186) in Model 1, but there was no significant association after further adjustment for comorbidity covariates in Model 2.

### Interaction Analyses and Subgroup Analyses

The results indicated a significant interaction between HVS category and sex (*p*s for interaction <0.05). We found no statistically significant effect modifications of age (highest HVS category × age: *p* = 0.927; medium HVS category × age: *p* = 0.720), and BMI (highest HVS category × BMI: *p* = 0.291; medium HVS category × BMI: *p* = 0.453).

Subgroup analyses based on sex and baseline diabetes diagnosis were shown in **Table 3**. The association between the highest HbA1c variability category (HVS=100) and functional limitation showed a similar pattern in male participants (β = 0.215, 95% CI: 0.089–0.342) and non-diabetes subgroup (β = 0.107, 95% CI: 0.022–0.193). However, this association between HbA1c variability and functional limitation in female subgroup lost significance in the fully adjusted model. We found no significant association of HbA1c variability with functional decline among individuals with diabetes.

**Table 3:** Subgroup analyses of association between HbA1c variability and functional limitation. (N=5544).

		β	SE	z	*p*	95% CI lower	95% CI Upper	OR (95% CI)
**Male (n=2195)**								
Model 1 ^a^	HVS=100	0.261	0.072	3.65	**<0.001**	0.121	0.401	1.298 (1.129, 1.493)
	HVS=50	0.149	0.056	2.65	**0.008**	0.039	0.260	1.161 (1.040, 1.297)
Model 2 ^b^	HVS=100	0.215	0.064	3.35	**<0.001**	0.089	0.342	1.240 (1.093, 1.408)
	HVS=50	0.055	0.049	1.13	0.257	-0.040	0.150	1.057 (0.961, 1.162)
**Female (n=3349)**								
Model 1 ^a^	HVS=100	0.120	0.049	2.47	**0.014**	0.025	0.215	1.127 (1.025, 1.240)
	HVS=50	0.083	0.036	2.28	**0.023**	0.012	0.153	1.087 (1.012, 1.165)
Model 2 ^b^	HVS=100	0.034	0.044	0.77	0.443	-0.052	0.119	1.035 (0.949, 1.126)
	HVS=50	0.050	0.033	1.54	0.123	-0.014	0.114	1.051 (0.986, 1.121)
**Diabetes (n=951)**								
Model 1 ^c^	HVS=100	0.136	0.075	1.80	0.073	-0.012	0.283	1.146 (0.988, 1.327)
	HVS=50	0.021	0.073	0.28	0.778	-0.122	0.163	1.021 (0.885, 1.177)
Model 2 ^d^	HVS=100	0.057	0.075	0.76	0.445	-0.089	0.203	1.059 (0.915, 1.225)
	HVS=50	0.009	0.070	0.13	0.895	-0.128	0.146	1.009 (0.880, 1.157)
**No diabetes (n=4588)**								
Model 1 ^c^	HVS=100	0.133	0.050	2.68	**0.007**	0.036	0.231	1.142 (0.996, 1.260)
	HVS=50	0.100	0.034	2.91	**0.004**	0.033	0.167	1.105 (1.034, 1.182)
Model 2 ^d^	HVS=100	0.107	0.043	2.47	**0.013**	0.022	0.193	1.113 (1.022, 1.213)
	HVS=50	0.056	0.030	1.84	0.066	-0.004	0.115	1.058 (0.996, 1.122)

HVS, HbA1c variability score. ^a^, adjusted for age, race, marital status, and mean HbA1c; ^b^, further adjusted for current smoking, BMI, depressive symptoms, cognition, hypertension, diabetes, cancer, lung disease, heart disease, stroke, arthritis and psychiatric disorder. ^c^, adjusted for age, sex, race, marital status, and mean HbA1c; ^d^, further adjusted for current smoking, BMI, depressive symptoms, cognition, hypertension, cancer, lung disease, heart disease, stroke, arthritis and psychiatric disorder. p values less than .05 are in bold.

### Sensitivity Analyses

The sensitivity analysis based on the CV and SD of HbA1c showed a similar association between HbA1c variability and functional limitation. As shown in **Table 4**, an incremental increase in the HbA1c-CV was associated with an increase in functional limitation (β = 0.630, 95% CI: 0.127–1.132) in the fully adjusted model. An incremental increase in the HbA1c-SD value was associated with an increase in functional limitation (β = 0.078, 95% CI: 0.006–0.150) in the fully adjusted model.

**Table 4:** Association between CV or SD of HbA1c variability and functional limitation score. (N=5544).

		β	SE	z	*p*	95% CI lower	95% CI Upper	OR (95% CI)
**Model 1**								
	HbA1c CV	1.263	0.264	4.79	**<0.001**	0.747	1.779	3.536 (2.111, 5.924)
	mean HbA1c	0.141	0.019	7.63	**<0.001**	0.105	0.177	1.151 (1.111, 1.194)
**Model 2**								
	HbA1c CV	0.630	0.257	2.45	**0.014**	0.127	1.132	1.878 (1.135, 3.102)
	mean HbA1c	-0.022	0.021	-1.06	0.288	-0.063	0.019	0.978 (0.093, 1.019)
**Model 1**								
	HbA1c SD	0.146	0.036	4.03	**<0.001**	0.075	0.218	1.157 (1.078, 1.244)
	mean HbA1c	0.135	0.021	6.51	**<0.001**	0.095	0.176	1.145 (1.100, 1.192)
**Model 2**								
	HbA1c SD	0.078	0.037	2.12	**0.034**	0.006	0.150	1.081 (1.006, 1.162)
	mean HbA1c	-0.028	0.023	-1.21	0.227	-0.072	0.017	0.972 (0.931, 1.017)

Model 1, adjusted for age, sex, race, marital status, and mean HbA1c; Model 2, further adjusted for current smoking, BMI, depressive symptoms, cognition, hypertension, diabetes, cancer, lung disease, heart disease, stroke, arthritis and psychiatric disorder. p values less than .05 are in bold.

## Discussion

To the best of our knowledge, this is the first study to examine the association between visit-to-visit HbA1c variability and functional limitations across a wide range of physical functional domains, and which analyzes data over a long-term 10-year follow-up period in a large population-based prospective cohort study. Potential confounders including mean HbA1c were comprehensively considered. We also considered the SD and CV of the annual mean HbA1c as an additional measure of glycemic variability.

Overall, this study found that HbA1c variability was associated with more difficulties in functional activities over time. After adjusting for multiple demographics, comorbidities, and mean HbA1c levels, HbA1c variability maintained an independent longitudinal association with more difficulties in functional activities. Sensitivity analyses using SD and CV of HbA1c instead of HVS did not materially change our results. This study provides further evidence for the detrimental effect of HbA1c variability and highlights the significance of steady glycemic control.

Going “beyond the mean HbA1c level” is an important focus of the current study. A cross-sectional study with the use of the ADA-recommended definition based on HbA1c measurement found that individuals with prediabetes had more physical function limitations than those with normoglycemia (11). A prospective cohort study revealed a U-shaped association of HbA1c levels and physical functioning impairment, and indicated that both high and low HbA1c levels were associated with a faster rate of decline in objectively measured physical functioning (18). In addition to average HbA1c measurement, previous meta-analysis showed that HbA1c variability was positively associated with adverse outcomes in micro- and macro-vascular outcomes and mortality independently of mean HbA1c, and HbA1c variability was more predictive of adverse outcomes than mean HbA1c in the majority of studies (20). The results of the present study add to those of previous studies, indicating that HbA1c variability was a superior predictor of functional decline over mean HbA1c.

The lack of a significant association of HbA1c variability with functional decline among individuals with diabetes may be due to a number of factors. A pooled analysis of two prospective population-based cohorts observed a significant association between long-term HbA1c variability and cognitive decline among the non-diabetic population but not among individuals with diabetes (23). The relatively small number of participants with diabetes in the present study (n = 951) may restrict the power to detect a positive association. Several studies have also reported that long-term HbA1c variability has a greater impact among individuals without diabetes, while short-term variability is a predictor among those with diabetes (38). The association of HbA1c variability with functional limitation among female subgroup lost significance in the fully adjusted model, suggesting that the effects of HbA1c variability may be explained by the preceding confounders including BMI, depressive symptoms, cognition, and comorbidities in females. Future studies are still needed to verify these observed associations.

To the best of our knowledge, this is the first study assessing the association between long-term HbA1c variability and functional decline that analyzes data from more than three physical functioning measurements over time. Many studies have been restricted to individuals diagnosed with diabetes, whereas others have included both diabetics and non-diabetics with stratification by diabetes diagnosis (20, 39). Our study further extends the findings of a significant association between long-term HbA1c variability and functional decline in a community-dwelling population. Recent systematic reviews have identified a range of potential risks associated with HbA1c variability but have had great difficulty in reaching clear conclusions (19, 20). This uncertainty may be due to the lack of a standard approach to summarizing HbA1c variability or agreement about how much might be clinically significant. Many studies use a relative measure (e.g., using quartiles of HbA1c variability), but this is difficult to compare across studies and even within the same study. The present study has mainly focused on the metrics of glycemic variability that are based on the HbA1c variability score (as it can be interpreted as the percentage of total HbA1c measures that vary by >0.5%), while omitting discussion of the more complicated computations to simplify the message, as a prerequisite for healthcare providers to be able to easily calculate and interpret in clinical practice.

The reasons for intraindividual variability in HbA1c are largely unknown. A previous study identified the patient characteristics associated with raised visit-to-visit glycemic variability in people with Type 2 diabetes, and thus the association of HbA1c variability with risk may not be a feature of the HbA1c variability per se but, rather, a marker of this baseline difference in patient characteristics (40). The current study adjusted comprehensively for baseline characteristics although we acknowledge that there could be residual confounding. Another study suggested that HbA1c variability is associated with the quality of care, indicated that intraindividual variability in HbA1c can be derived from poor quality of care or poor compliance with medical recommendations (41).

The pathophysiological mechanisms involved in the observed association between visit-to-visit glycemic variability and functional limitations remain unclear. Glycemic variability is a measure that accounts for the amplitude, frequency, and duration of glycemic oscillations around the average blood glucose level and an integral component of glucose homoeostasis (19, 42). Glycemic variability may be associated with functional disability through mechanisms associated with oxidative stress, chronic systemic inflammation, extremes of blood glucose, decreased muscle strength, lower muscle quality, and accelerated loss of muscle mass (7, 8, 38, 42–45). Oxidative stress is suggested to explain the association between short-term glycemic variability and adverse outcomes (41), but it is not clear whether this is increased in patients with high visit-to-visit HbA1c variability. Glycemic variability can represent the presence of excess glycemic excursions and, consequently, the risk of hyperglycemia or hypoglycemia (43). High concentrations of glucose might lead to systemic, chronic inflammation, which is part of a multifactorial process that eventually results in frailty and disability (8, 45). Further studies are necessary to clarify the mechanisms underlying the association between glycemic variability and functional limitations.

The findings of the present study should be considered in the context of some potential limitations, including the observational study design which does not allow for casual inference and self-reported measures of comorbidities, which may underestimate the true prevalence of these conditions. Difficulties in physical functioning were also based on self-reported measures. Although self-reports provide valuable information about the person’s own perception of their functioning in the living environment, replication using objective physical performance measures would alleviate concerns regarding potential self-reported bias. Although we adjusted for many potential confounding factors, there remains the possibility that residual confounding factors were not measured in this association, which could have influenced the variability observed in the study.

Despite these limitations, the strength of the present study is the use of a large, representative, longitudinal cohort of middle-aged and community-dwelling elderly and large data of multiple visit-to-visit HbA1c measures, enabling us to accurately calculate long-term HbA1c variability over a long-term 10-year follow-up period. The outcome measure was based on difficulty in 17 physical functioning tasks across different physical functional domains, covering not only ADLs and IADLs but also other clinically relevant disability domains, such as mobility (e.g., walking several blocks), and general physical activities (e.g., stooping, bending, and pulling a large object). Many previous studies have focused on single disability domains or items; however, it is common for older people to have difficulties in multiple areas of physical functioning (35). This comprehensive assessment allowed us to explore the association between HbA1c variability and composite functional limitations across multiple physical functional domains.

## Conclusion

In conclusion, data from a population-based sample of US adults indicate the association between glycemic variability, as measured by variability score in visit-to-visit HbA1c over time, and the number of physical functioning difficulties independent of mean HbA1c in individuals aged over 50 years. This association remained significant even after adjusting for sociodemographic and clinical factors. Further well-controlled randomized controlled trials are needed to establish glycemic variability as an independent risk factor for functional decline and diabetes complications and to confirm whether strategies to reduce glycemic variability in HbA1c can effectively reduce the incidence or progression of physical functioning impairment. Future studies are also needed to investigate the potential of using HbA1c variability in assessing risk in older people and to inform optimal approaches to achieving a safe and stable glycemic level.

## Authors Contributions

DS: Conception and design, acquisition of data, analysis, drafting. SSW, JWS, HPWand QS: Conception and design, analysis and interpretation of data, critical revision of the manuscript. All authors contributed to the article, approved the version to be published and agreed to be accountable for all aspects of the work.

## Data Availability Statement

The datasets used and/or analyzed during the current study are available from the corresponding author on reasonable request.

## Ethics Statement

All participants provided informed consent, and the HRS is approved by the Institutional Review Board at the University of Michigan.

## Funding

This study was funded by the National Key Research and Development Program of China (Grant Number: 2020YFC2006505) and the National Natural Science Foundation of China (Grant Number: 31900785). The study funders were not involved in the design of the study, collection, analysis, and interpretation of data and in writing the manuscript.

## Conflict of Interest

The authors declare that the research was conducted in the absence of any commercial or financial relationships that could be construed as a potential conflict of interest.

## Publisher’s Note

All claims expressed in this article are solely those of the authors and do not necessarily represent those of their affiliated organizations, or those of the publisher, the editors and the reviewers. Any product that may be evaluated in this article, or claim that may be made by its manufacturer, is not guaranteed or endorsed by the publisher.

## Abbreviations

ADLs, activities of daily living; DBS, dried blood spot; HRS, Health and Retirement Study; HVS, HbA1c variability score; IADLs, instrumental activities of daily living; SD, standard deviation; CV, coefficient of variation.
